# Supplementary material for: Metabolic impacts of cordycepin on hepatic proteomic expression in streptozotocin-induced type 1 diabetic mice
Source: PLoS One. 2021 Aug 13;16(8):e0256140. doi: 10.1371/journal.pone.0256140 (PMC8363009; doi:10.1371/journal.pone.0256140)
Supplement: S1 Table — (PDF) [file pone.0256140.s001.pdf]

S1 Table. Protein identification and functional classification of unique proteins in normal mice (N), normal mice treated with cordycepin (N+COR), and diabetic mice (DM).

Accession No.	Gene name	Protein name	Peptide sequence	Mass (Da)	Biological process
Normal mice (N):					
gi|206409	PRP	Proline-rich protein	AKPVQGTATHVEKSPK	558,483	Unknown
gi|189181712	Cfap161 RGD1310371 rCG_24783	Cilia and flagella-associated protein 161	NKSNTMLDISKPIAVDTRAVEQAMGINT	34,500	Unknown
gi|26338664	Fndc10 B930041F14Rik	Uncharacterized protein	GPRDGRRAPVLSQPR	24,305	Unknown
Normal mice treated with cordycepin (N+COR):					
gi|454527343	Dst Bpag1 Macf2	Dystonin (Bullous pemphigoid antigen 1)	YELSAVQLEKASSEEKAR	834,218	axonogenesis, cell adhesion
gi|1040281672	QTRT1 A6R68_18159	Queuine tRNA-ribosyltransferase catalytic subunit 1	MAAAGSPASLESAPRILR	45,805	tRNA-guanine transglycosylation
gi|852778012	Sipa1l3	signal-induced proliferation-associated 1-like protein 3	VPRPTKPHKPPGSIGLCGGR	163,104	positive regulation of GTPase activity
gi|852787791	Mgarp	protein MGARP	SGQPARPVRASSSGPSPR	24,787	anterograde axonal transport
Diabetic mice (DM):					
gi|27465603	Akr1b8 aldose reductase-like	Aldose reductase-like protein	MPIVGLGTWKSMPNQVKEAVK	36,191	Unknown
gi|1040286095	A6R68_21115	Uncharacterized protein	PSPKGQRDGDPTYAEAYAMK	43,024	viral process
gi|914914450	ZMAT3	Zinc finger matrin-type 3	RPSSSPPMSVATRSSGTLQLPPQK	31,935	Unknown
gi|156447039	Calml4	Calmodulin-like protein 4	QEDPKKEILLAMLMADKEK	12,354	Unknown
gi|852769487	Ccdc180	coiled-coil domain-containing protein 180	KTSYLMQPDVYR	202,709	Unknown
gi|344248806	I79_007809	Peroxidase	PMRRCLQALVLCFSLGAMAVVASKPGAGCPSR	165,365	response to oxidative stress
gi|50403743	Gdf7 Gdf-7	Growth/differentiation factor 7	GAQGSGGGGGGGGGGGGGGGGGGAGR	47,891	activin receptor signaling pathway
gi|344256598	I79_017663	Protein NipSnap-like 2	MWLWLCFRRVTSSSHR	12,840	Unknown

Accession No.	Gene name	Protein name	Peptide sequence	Mass (Da)	Biological process
gi|1040154546	A6R68_05356	Uncharacterized protein	RSLMAITSMNWDTIPTR	16,206	Unknown
gi|852738019	Ears2	Glutamyl-tRNA synthetase (Probable glutamate--tRNA ligase, mitochondrial)	LELLKKEALRSYQTPR	56,355	glutamyl-tRNA aminoacylation
gi|351695934	GW7_07125	Ras-related protein Rab-39A	FRVLLLGDAGVGK	10,313	Unknown
gi|852728663	Tbc1d30	TBC1 domain family member 30	RQGGGGGGGGGGGVGTILSNVLK	81,890	Unknown
gi|226423933	Col3a1	Collagen alpha-1(III) chain	GLAGPPGMPGPRGSPGPQGIKGESGK	138,943	integrin-mediated signaling pathway, negative regulation of immune response, transforming growth factor beta receptor signaling pathway
gi|351703107	GW7_20741	Uncharacterized protein	SCPARPGDLVPAPAR	9,942	Unknown
gi|155369299	Pard6g	Partitioning defective 6 gamma	GDGGLHSSGRESNGSIHR	42,174	regulation of cellular localization
gi|1040274154	A6R68_18670	Uncharacterized protein	FGWAGPGSPAPGR	43,880	Unknown
gi|852755936	Fam186b	protein FAM186B	STEALESKR	100,615	Unknown
gi|549440246	RAG1	V(D)J recombination-activating protein 1	EAMKSSELMXEMGGILR	74,251	B cell differentiation, V(D)J recombination
gi|341942266	Pygl	Glycogen phosphorylase, liver form	VIFLENYR	97,463	5-phosphoribose 1-diphosphate biosynthetic process, glycogen metabolic process
gi|852795427	Tnfrsf11b	Tumor necrosis factor receptor superfamily member 11B	TRKMCKSSEQLLK	46,115	apoptotic process
