# Supplementary material for: Metabolic impacts of cordycepin on hepatic proteomic expression in streptozotocin-induced type 1 diabetic mice
Source: PLoS One. 2021 Aug 13;16(8):e0256140. doi: 10.1371/journal.pone.0256140 (PMC8363009; doi:10.1371/journal.pone.0256140)
Supplement: Attachment — Submitted filename: Response to Reviewer-PLOS ONE.docx [file pone.0256140.s002.docx]

**Response to the reviewer’s comments**

Dear Editor,

Thank you for giving us an opportunity to revise our manuscript entitled “*Metabolic impacts of cordycepin on hepatic proteomic expression in streptozotocin-induced type 1 diabetic mice*”. We appreciate the insightful and constructive comments made by the reviewers, and have made revisions. Please find attached the revised manuscript. The manuscript has not been published in any other journal.

**Journal Requirements:**

***1:*** *Please ensure that your manuscript meets PLOS ONE's style requirements, including those for file naming. The PLOS ONE style templates can be found at*

[*https://journals.plos.org/plosone/s/file?id=wjVg/PLOSOne_formatting_sample_main_body.pdf*](https://journals.plos.org/plosone/s/file?id=wjVg/PLOSOne_formatting_sample_main_body.pdf) *and*

[*https://journals.plos.org/plosone/s/file?id=ba62/PLOSOne_formatting_sample_title_authors_affiliations.pdf*](https://secure-web.cisco.com/1GWGTsdZYtI3aNK0Q1HFrJiSwnT-x6A-mB_h_iA9cjnbVkwvDy5H_3cxQPl-av-KfKYXrWclKv1bICqdVDTQCAUYoPL1DegJuGmX6-HMw2hoPMlklhFVVuM1pILdtXHhrNr5bghVv_WRoLTKJkjmvM6nGhurr1mFEbENBeyexvM7KaainBx4k46VnRVQoIGQsWmWkEvK8D7WeMu_M0nLgkeTfVYcFqsZoAGVa1GSwjWcMx8kzY5KCI69xun953d3SgNBzAffiiy-IqYxmS0FGeQCj5lHyr8GJr872DUNXZdU/https%3A%2F%2Fjournals.plos.org%2Fplosone%2Fs%2Ffile%3Fid%3Dba62%2FPLOSOne_formatting_sample_title_authors_affiliations.pdf)

**Response:**

This manuscript has been revised according to the journal style requirements (please see the revised manuscript).

***2:*** *Please amend your Methods section to state the method of animal sacrifice used. If the method of sacrifice was injection of sodium pentobarbital, please change the word "anesthetized" (line 122) to "euthanized”.*

**Response:**

We have checked and changed the word “anesthetized” to “euthanized” on line 150 of the material and method as read as follow:

**At the end of the experimental period, all animals were euthanized with 60 mg/ml of pentobarbital sodium.**

***3:*** *Thank you for including your ethics statement:  "The ethical guidelines about the proper care and use of laboratory animals, outlined by the Kasetsart University Research and Development Institute, Kasetsart University, Thailand (ID: ACKU60-SCI-014), were followed.".*

*Please amend your current ethics statement to confirm that your named ethics committee specifically approved this study.*

**Response:**

We have also added the following sentence on lines 129-132 of the material and method:

**The research conducted adhered to the Guidelines for the Care and Use of Laboratory Animals. The ethics committee of Kasetsart University Research and Development Institute, Kasetsart University, Thailand, approved this study (Approval No. ACKU60-SCI-014).**

***4:*** *We note that you have stated that you will provide repository information for your data at acceptance. Should your manuscript be accepted for publication, we will hold it until you provide the relevant accession numbers or DOIs necessary to access your data. If you wish to make changes to your Data Availability statement, please describe these changes in your cover letter and we will update your Data Availability statement to reflect the information you provide.*

*For additional information about PLOS ONE submissions requirements for ethics oversight of animal work, please refer to* [*http://journals.plos.org/plosone/s/submission-guidelines#loc-animal-research*](http://journals.plos.org/plosone/s/submission-guidelines#loc-animal-research)

*Once you have amended this/these statement(s) in the Methods section of the manuscript, please add the same text to the “Ethics Statement” field of the submission form (via “Edit Submission”).*

**Response:**

Thank you for your kind reminder.

**Response to Reviewer #1 comments:**

**Comment 1:** *STZ: how was administered? Duration of administration?*

**Response:**

We have also added the following sentences to the material and method section (lines 134-138):

**The STZ was prepared by dissolving in a citrate buffer (0.1 M, pH 4.5) at a dosage of 200 mg per kg of body weight. The normal mice received only the same volume of citrate buffer. After 36 h under observation, plasma glucose level was detected and mice with plasma glucose levels that exceed 250 mg/dl were considered as diabetes.**

**Comment 2:** *STZ e cordycepin were administered contemporanely?
Some specificiantions regarding Cordycepin are necessary:*

**Response:**

We have also added the following sentences to the material and method section (lines 142-144):

**Mice in each treatment were intraperitoneally administrated for 14 days once per day. Cordycepin was dissolved in distilled water at a dose of 24 mg/kg/day, according to Ma *et al*. [17].**

**Comment 3:** *Cordycepin administred was extracted from Cordyceps or was syntetised or purchased from chemical company?*

**Response:**

We have also added the following sentence on lines 121-124 of the material and method section:

**Cordycepin, streptozotocin (STZ), pentobarbital sodium, and TCA cycle standard samples (oxaloacetate, alpha-ketoglutarate, and citrate) were purchased from Sigma (St. Louis, USA).**

**Comment 4:** *Please show the structure of the Cordycepin*

**Response:**

**We have showed the chemical structure of cordycepin as shown in Fig1 on introduction section (line 118).**

**Comment 5:** *How was dissolved Cordycepin and what vehicle was used?*

**Response:**

We have also added the following sentence on lines 142-144 of the material and method section:

**Cordycepin was dissolved in distilled water at a dose of 24 mg/kg/day, according to Ma *et al*. [17].**

**Comment 6:** *How was administered Cordycepin: intraperitoneal or other?*

**Response:**

We have also added the following sentence on lines 142-144 of the material and method section:

**Cordycepin was dissolved in distilled water at a dose of 24 mg/kg/day, according to Ma *et al*. [17].**

**Comment 7:** *What is the frequency of administration one dose or daily or other?*

**Response:**

We have also added the following sentence on lines 142-144 of the material and method section:

**Cordycepin was dissolved in distilled water at a dose of 24 mg/kg/day, according to Ma *et al*. [17].**

**Comment 8:** *Lines 117-120 Blood glucose level was monitored once a week during the experimental period. Body weight and food consumption were monitored daily by weighing the animal at 11:00 hrs; the food intake of each animal was measured by weighing the remaining chow.*

*If glucose was measured once a week What glucose measurement is shown in Table 1? Are these final measures?*

*Morerover, if glucose was measured once a week, at least three measurements per animal are available, while body weight and food consumption measurements are recorded daily (14 measures are available). It would be more complete to show all in a chart and add final data in the text.*

**Response:**

We have also added the following sentence on lines 220-230 of the results section:

**As shown in Fig 2A, the streptozotocin-induced diabetic mice (DM) mice showed significant increase on fasting blood glucose levels at 0, 7 and 14 days. However, the cordycepin treatment on DM mice did not show significant effect to fasting blood glucose level at all the time intervals.  The difference in body weights among all of the groups in the initial days was not significant. After 7 days, the body weights of the DM and DM+COR were lower than that of the N group (Fig 2B). As shown in Table 1, DM exhibited a low final body weight, an increased food intake, and an increased water intake. Nevertheless, cordycepin treatment of the diabetic mice (DM+COR) significantly decreased the body weight, food intake and water intake when compared to DM. On the other hand, cordycepin treatment did not affect the blood glucose levels and liver index when compared to the DM group.**

**Comment 9:** *I would suggest to perform other serum measurements such as transaminases or inflammation markers.*

**Response:**

The propose of our study was to evaluated the effect of cordycepin on hepatic metabolize and protein expression in STZ-induced diabetic mice, so we did not examine the inflammatory marker in this study. However, the previous study indicated that the maximum tolerance dose of cordycepin is 3600 mg/kg and did not show significant effect on body weight and food intake (Ma et al., 2015).

**Comment 10:** *Finally a list proteins shown to be differentially expressed by the Venn diagram in figure 2 should be included as supplementary data.*

**Response:**

The supporting information of a unique protein list in N group, N+COR group and DM group have been shown in S1 Table. Please see the additional/revised S1 table.

We are grateful to all reviewers for their constructive comments and consider that the changes to the text and clarifications we have made have improved the manuscript.

Yours sincerely,

Wirasak Fungfuang
